# Neodymium-Mediated Coordinative Chain Transfer Polymerization of Isoprene in the Presence of External Donors

**DOI:** 10.3390/molecules28217364

**Published:** 2023-10-31

**Authors:** Aiwu Ding, Liang Fang, Chunyu Zhang, Heng Liu, Xuequan Zhang, Jianhe Liao

**Affiliations:** 1 School of Life Sciences, Hainan University, Haikou 570228, China; dingaw@163.com; 2 China Hainan Rubber Industry Group Co., Ltd., Haikou 570106, China; 3 Key Laboratory of Rubber-Plastics, Ministry of Education, Shandong Provincial Key Laboratory of Rubber-Plastics, Qingdao University of Science & Technology, Qingdao 266061, China; cyzhang@qust.edu.cn (C.Z.); hengliu@qust.edu.cn (H.L.); xqzhang@qust.edu.cn (X.Z.); 4 School of Materials Science and Engineering, Hainan University, Haikou 570228, China

**Keywords:** coordinative chain transfer polymerizations, neodymium, polyisoprene, synthetic rubber, external donor

## Abstract

Nd-based polydiene elastomers, including NdIR and NdBR, are regarded as indispensable key raw materials in preparing green tires with excellent performance capabilities, but their wide application is still limited by the relative higher cost of Nd precatalysts. Nd-mediated coordinative chain transfer polymerization (CCTP) of diene provides an effective strategy to reduce the precatalyst cost, because this method involves very high atom economy, i.e., each Nd molecule can generate multiple polymer chains. Nevertheless, all possible factors that could influence such CCTP behaviors are still mostly unexplored to date. In this report, the basic chemistry on the influence of external donors on the overall CCTP behaviors of isoprene was established for the first time. It was found that increasing the amount of external donors had a negative influence on the chain transfer efficiencies, resulting in gradually decreasing atom economies. Catalyst addition order studies revealed that the coordination of donors with cationic Nd active species, rather than alkylaluminium CTAs, contributed mostly to such decreased efficiencies. Moreover, it was found that when the ratio of donors and Nd compounds was higher than 1.0, the CCTP behaviors were corrupted, resulting in polymers with broad distributions, as well as resulting in low atom economies; nevertheless, when the ratio was lower than 0.5, the system still displayed CCTP characteristics, implying that the critical ratio for maintaining the CCTP was 0.5. Additionally, when such a ratio was 0.01, the high atom economy was almost the same as donor-free CCTP systems. Detailed kinetic studies at such a ratio demonstrated that the donor-contained system proceeded in a well-controlled manner, as concluded from the good linear relationship between the *M*_n_ of the PIps against the polymer yields, as well as the good linearity between the *M*_n_ against the (IP)/(Nd) ratios. Such maintained CCTP properties also allowed for seeding two-step polymerizations to prepare diblock copolymers with precisely controlled molecular weights. Expanding the types of donors to more phosphine, oxygen, and nitrogen containing compounds showed that they also affected the CCTP behaviors depending on their steric and electronic properties.

## 1. Introduction

Coordinative chain transfer polymerization (CCTP) represents a modern strategy to access polymers in a well-controlled manner [1,2,3,4,5,6,7,8,9,10,11,12,13,14,15,16,17,18,19,20,21,22,23,24]. A typical CCTP catalytic system generally comprises a transition-metal or lanthanide-based precatalyst and a chain transfer agent (CTA), which is usually in the form of a main group metal alkyl. During polymerization, polymer chains that are originally propagated from the precatalyst can be reversibly transferred to the CTA, and such a transfer rate is so rapid (vs. the chain propagation rate) that monomers appear to synchronously propagate on the CTA, which eventually results in polymer products with precisely controlled molecular weights and narrow molecular weight distributions, that parallels anionic living polymerizations. Another CCTP characteristic is that multiple chains can be generated per molecule of the precatalyst, demonstrating high atom economy and showing a striking contrast to traditional anionic living polymerization systems, where one metal can only generate one chain. Because of these unique properties, CCTP has witnessed tremendous growth during the past two decades.

Neodymium (Nd)-based CCTP of 1,3-butadiene and isoprene is of great importance for the current synthetic rubber industry, because the produced neodymium-based polybutadiene (NdBR) and polyisoprene (NdIR) are regarded as indispensable key raw materials in preparing green tires with excellent performance capabilities [25,26]. Although NdBR and NdIR have realized industrialization by using traditional catalytic systems, their relatively higher precatalyst cost compared to other types of BR (such as NiBR, CoBR, TiBR, etc.) and IR (such as TiIR, etc.) still limits their widespread application. By contrast, a CCTP strategy can overcome such a shortage, because it demonstrates a highly atom-economic characteristic, that is, one Nd atom can produce multiple polydienyl chains, which therefore serves as an effective strategy to reduce the cost of a Nd-based precatalyst [27,28,29,30,31,32,33,34,35,36,37,38]. Based on this consideration, much of our recent research has looked to explore any factors that could have an influence on CCTP behaviors [28,29,30,34,36,39]. Of all the possible factors, heteroatom-based external donors are of great importance, because they are often used to disassociate the clustered structure of the neodymium precatalyst or to facilitate the formation of active species [40,41,42,43,44,45]. Additionally, for the whole continuous production line of NdIR and NdBR, impurities in the polymerization system, no matter whether they are from the recovered solvent or from the monomer, also contain heteroatoms. Nevertheless, to date, their detailed influence on CCTP behaviors is still unclear. Will they facilitate or slow down CCTP behavior? If they slow down CCTP behavior, what is the possible mechanism? Can we find a critical point (i.e., the maximum amount of external donors), below which the CCTP characteristic can be maintained to guarantee the atom-economic feature? Based on this consideration, in this report, we will try to elucidate the influence of various external donors on the Nd-mediated CCTP of isoprene.

**Scheme 1:**
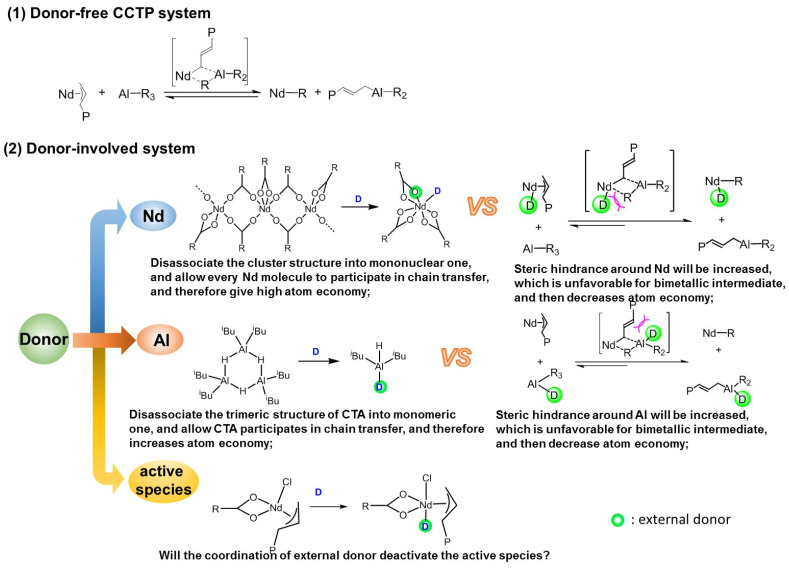
Illustrative profiles for donor-free and donor-involved CCTP system.

During CCTP, the key chain transfer step proceeds via a bimetallic intermediate (Scheme 1(1)), therefore, it can be safely concluded that any factors that can affect the formation of the bimetallic intermediate would have a big influence on the overall CCTP behaviors. Due to the presence of several electron lone pairs, heteroatom-based external donors could easily coordinate to the Lewis acidic metal centers, no matter whether it is for the transition-metal or lanthanide-based precatalyst, or the main-group metal-based CTA. Therefore, external donors are anticipated to have a big influence on the formation of the bimetallic intermediate and, thereafter, on the overall CCTP behaviors. For the Nd-mediated CCTP of isoprene, the influence of external donors is very complicated because of the presence of the following two paradox relationships (Scheme 1(2)). Firstly, for the Nd precatalyst, external donors are capable of disassociating the original clustering multi-nuclear structure into a mononuclear one, which is beneficial for CCTP because every Nd molecule can participate in the chain transfer process, resulting in higher atom economies; nevertheless, after being coordinated by external donors, steric hindrance around the Nd will be increased, which will slow down the chain transfer reaction because the steric congested structure is unfavorable for the formation of the bimetallic intermediate. The slowed chain transfer reaction will decrease the atom economies. So, for those two possible outcomes, which one will be the primary one (paradox one)? Secondly, for alkylaluminium CTAs, such as diisobutylaluminium hydride (DIBAH), they often exist as a trimeric structure, which therefore only allows one third of the DIBAH to participate in CCTP [31,46]; however, the introduction of an external donor can break the trimeric structure into a monomeric one, allowing every DIBAH molecule to participate in CCTP and, hence, revealing high atom economy. Nonetheless, after being coordinated by external donors, steric hindrance around the aluminum center will also be enhanced, which again has disadvantages for the bimetallic intermediate. So, again for this paradox, which one will be operative (paradox two)? Additionally, for cationic active species, they can also be coordinated by donors, which also poses a risk of deactivation. Based on these analyses, this research will try to figure the situation out and also put forward possible explanations.

## 2. Results and Discussion

Before exploring the influence of external donors, an optimum CCTP catalytic system needed to be screened first. It has been previously concluded that DIBAH is the most efficient CTA for Nd-mediated CCTP systems [31,46]. Additionally, such DIBAH usually adopts a trimer structure in alkane solvents, which serves as a good candidate for showing the influence of donors, because it disassociates into a monomeric structure after donor coordination. Therefore, DIBAH involving a CCTP catalytic system of NdV/DIBAH/SiMe_2_Cl_2_ was intentionally selected herein, to elucidate the overall influence of external donors on CCTP behaviors. A phosphine-containing donor of tri(*tert*-butyl)phosphine (T*t*BP) was intentionally selected as a representative to achieve the optimized polymerization conditions, because it possesses a very sterically bulky nature.

High atom economy is the primary goal in this study, because only a higher atom economy can guarantee the low cost of the precatalyst. Therefore, the study commenced by exploring the influence of (T*t*BP)/(Nd) ratios on the polymerization performance. As can be seen from the data in Table 1, for the T*t*BP-free system, the polymerization displayed the anticipated typical CCTP characteristics, i.e., extremely narrow molecular distributions, high atom economies, etc. Increasing the (T*t*BP)/(Nd) ratios from 0.01 to 0.1 and 0.5 had little influence on the CCTP characteristics; narrow molecular weight distributions (1.66–1.70) were still obtained under these conditions. However, when further increasing the (T*t*BP)/(Nd) ratios to 1.0 and 3.0, much broader molecular weight distributions were obtained (2.45 and 2.95), suggesting that the chain transfer reactions in these two cases were not reversible anymore, which also hinted at the corruption of the CCTP characteristics. Based on these studies, it could be concluded that a maximum ratio of (T*t*BP)/(Nd) = 0.5 is the critical point for guaranteeing the CCTP behaviors if a high atom economy is expected. Slowed down chain transfer rates, also called decreased chain transfer efficiencies, can also be manifested from the gradually increased molecular weights and decreased *N*_p_ values. For the T*t*BP-free system, a molecular weight of 1.85 × 10^4^ Dalton was obtained. When gradual increasing the (T*t*BP)/(Nd) ratios from 0.01 to 3, the molecular weights of the obtained polyisoprene monotonously increase from 1.95 × 10^4^ to 3.69 × 10^4^. In the case of the *N*_p_, which is a key value for assessing the atom economy of the CCTP system, a gradually decreasing trend from 3.67 to 1.84 was demonstrated, showing that the introduction of external donors always has a negative influence on the atom economies. Nevertheless, when (T*t*BP)/(Nd) = 0.01, the *N*_p_ values only slightly reduced from 3.67 to 3.50, implying a negligible negative influence. Based on this consideration, the following studies were all carried out at such a ratio to maintain a high atom economy, as in the donor-free system.

**Figure 1:**
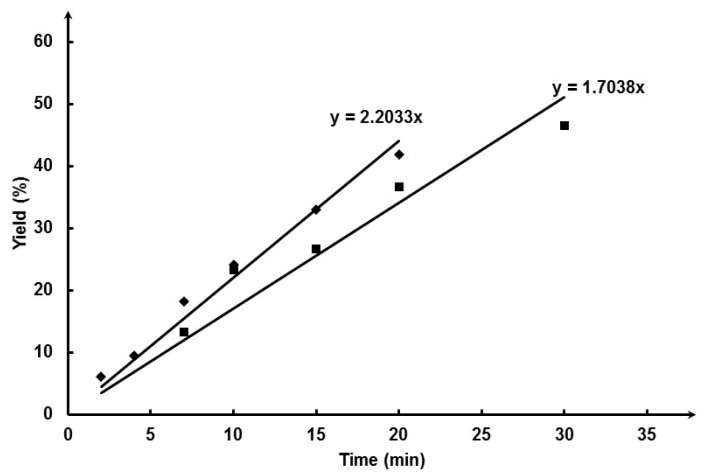
Plots on polymer yield versus time in the presence (◆) or absence (■) of T*t*BP.

Regarding the influence of donors on the catalytic activities of the present CCTP system, no decrement in the PIp polymer yields were detected when varying the (T*t*BP)/(Nd) ratios from 0.01 to 3, implying that the generated active species could tolerate such concentrations of heteroatoms. Additionally, the introduction of 0.01% T*t*BP could enhance the polymerization propagation rates. As in the plots on the polymer yields versus time shown in Figure 1, the slopes increased from 1.7038 to 2.2033, demonstrating the obviously increased polymerization rate.

The decreased transfer efficiencies in Table 1 are highly likely related to the coordination with the Nd or Al centers, which increased the steric bulkiness and, thus, resulted in slower chain transfer reaction rates (Scheme 1(2)). Such a result provided a clear answer to the two paradox relationships proposed in the introduction section. That is, although the donors were capable of disassociating clustered Nd compound or trimeric DIBAH, they did not improve the performance of the overall CCTP characteristics and also the atom economy. In contrast, a decreased atom economy because of the coordination with the Nd or Al center was concluded to be primary, due to the increased steric congestion around the metal centers that decreased the chain transfer rates. As with the mechanism shown in Scheme 1, a chain transfer generally occurs through a transmetalation reaction via a heterobimetallic intermediate. For the donor-free CCTP system, due to the presence of excessive alkylaluminium (AlR_3_)-based CTA, such a transmetalation process can occur facilely, resulting rapid polyisoprenyl and alkyl exchange between the two metal centers. Whereas for the donor-involved CCTP system, when the Nd atom or Al atom was coordinated by donors (Scheme 1(2)), the steric crowdedness around the corresponding metals increased significantly and, due to the steric repulsion between the polyisoprenyl and alkyl groups, the bimetallic intermediate could not be formed as easily as in the donor-free system, thus giving rise to slowed down chain transfer rates. Despite this, when (T*t*BP)/(Nd) < 0.5, the slower chain transfer rates were still much faster than the chain propagation rate (*k*_tr_ >> *k*_p_) to guarantee the CCTP characteristic, as evidenced by the narrow molecular weight distributions (1.66–1.70), otherwise broader ones would have resulted.

**Figure 2:**
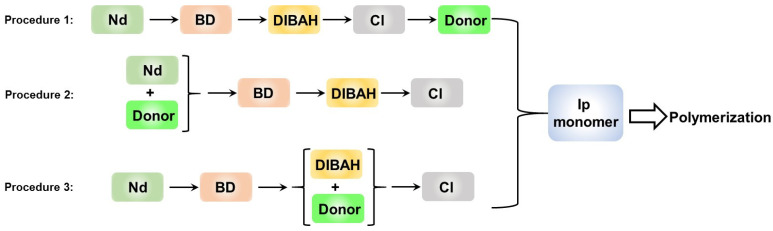
Procedures for catalyst preparation during the aging stage.

Now that it has been established that the coordination of the external donors to the metal centers can decrease the chain transfer rates, it is important to distinguish which kind of coordination, i.e., whether with the Nd or Al atom, plays the more primary role. The answer to such a question can be easily inferred by investigating the catalyst addition order on the overall CCTP behaviors (Figure 2). As can be seen from the data shown in Table 2, the studies were carried out by premixing the donors with the Nd precatalyst (entry 2, Table 2, or procedure 2 in Figure 2) or the Al cocatalyst (entry 3, Table 2, or procedure 3 in Figure 2) and the subsequent reaction with other components during the aging stage. In a benchmark reaction for comparison, a donor was directly mixed with the generated active species (entry 1, Table 2, or procedure 1 in Figure 2). It was found that when premixing T*t*BP with an alkylaluminium cocatalyst, PIp products with much higher molecular weights (2.21 × 10^4^ vs. 1.95 × 10^4^ Dalton) and an *N*_p_ with a much smaller value (3.08 vs. 3.50) were obtained, implying its inconsistency with the benchmark reaction. Nevertheless, for the reaction from premixing T*t*BP with NdV, the formed active species provided very similar results to the benchmark CCTP system, no matter what the molecular weight (1.95 × 10^4^ vs. 1.95 × 10^4^ Dalton) and microstructures (96.9% vs. 96.8% 1,4-contents) of the PIp products or the *N*_p_ values (3.49 vs. 3.50) were, illustrating that the donor of the T*t*BP was mainly coordinated with the Nd atom to affect the polymerization. This result is reasonable when considering the cationic Nd-based active species, which revealed a higher binding affinity with the Lewis base donors than neutral alkylaluminium compounds.

After making clear the mechanism of how external donors of T*t*BP influenced the polymerization behavior, other polymerization conditions, including the (Al)/(Nd) and (Cl)/(Nd) ratios, were next investigated in order to provide a whole map on the donor-involved CCTP system. All of these studies were conducted at (T*t*BP)/(Nd) = 0.01 to maintain a high atom economy, as concluded above. As shown in the results summarized in entries 1–5 in Table 3, lower (Al)/(Nd) ratios could not fully initiate the polymerizations, resulting in relatively lower monomer conversions. For instance, when (Al)/(Nd) = 15 and 20 were applied, monomer conversions were achieved for 71% and 80%, respectively. Additionally, under such low (Al)/(Nd) ratios, the formed active species were not structurally uniform, resulting in relative broader dispersities (2.46 and 2.90). Increasing the (Al)/(Nd) ratio to 25 could achieve full monomer conversion, as well as narrow molecular weight distributions (1.66), suggesting the formation of uniform active species, i.e., the successful construction of CCTP behaviors. Further increasing the (Al)/(Nd) ratio to 30 could further enhance the chain transfer efficiencies, as revealed by the decreasing molecular weights from 1.94 × 10^4^ to 1.53 × 10^4^. Nevertheless, such a condition also induced the heterogeneity of the active species, perhaps due to the formation of neodymium–hydride clusters, which have been observed previously in the literature [34]. Therefore, an optimal (Al)/(Nd) ratio of 25 was obtained herein. It is of note that such a ratio was comparatively higher than that for donor-free systems ((Al)/(Nd) = 10~20) in order to achieve CCTP behaviors [31,34], which might also be due to the coordination of T*t*BP with the Nd or Al centers that slowed down the chain transfer reactions.

Besides, chloride compounds also played a pivotal role in governing the CCTP performance in the presence of T*t*BP. As shown in entries 6–10 in Table 3, when low (Cl)/(Nd) ratios of 0.5 and 1.0 were applied, insufficient active species were formed, which resulted in relatively lower polymer yields of 60% and 73%. The optimal (Cl)/(Nd) ratio was observed to be 2.0, under which condition a quantitative monomer conversion and narrow molecular weight distributions were obtained. Further increasing the (Cl)/(Nd) ratio to 3.0 and 5.0 resulted in heterogeneous catalytic systems as well, which hence resulted in broader molecular weight distributions that deviated from CCTP behaviors. Despite these results, it should be noted that the presence of T*t*BP donors had little influence on the formation of Cl-containing active species, because such a (Cl)/(Nd) = 2.0 ratio was the same as previously reported for donor-free CCTP systems.

After acquiring the optimal conditions from T*t*BP-involved CCTP studies, the types of external donors were then expanded to more phosphine (P), oxygen (O), and nitrogen (N)-containing counterparts, and each of them was structured with different alkyl or aryl groups. Representative phosphine compounds, including tri(*n*-butyl)phosphine (TnBP), tri(*tert*-butyl)phosphine (T*t*BP), tri(*n*-octyl)phosphine (TOP), triphenylphosphine (TPP), and diphenylmethylphosphine (DPMP). And for oxygen and nitrogen containing compounds, diethylether (DEE), dibutylether (DBE), phenylmethylether (PME), 1-hexanol (HO), undec-10-en-1-ol (UO), triethylamine (TEA), trioctylamine (TOA), diisopropylethylamine (DIEA), diisobutylamine (DIBA), and phenylethylamine (PEA), were selected. As can be seen from the results shown in Table 4, the types of donors and their structures also posed obvious influences on the CCTP behaviors. For phosphine compounds, T*n*BP, TOP, and DPMP-involved systems resulted in a slightly lower polymer molecular weight than phosphine-free systems, which hinted at slightly increased chain transfer rates. Whereas for T*t*BP-involved systems, a slightly higher molecular weight was observed. Such distinctly different influences from phosphine donors might originate from their different steric sizes. For T*t*BP, its bulky nature prohibited chain transfer reactions after its coordination with the metal centers, whereas for the former three compounds, their coordination could dissociate the Nd cluster or the DIBAH trimer structures, which resulted in the facilitation of chain transfer rates and, therefore, smaller molecular weights. For nitrogen- and oxygen-containing donor-involved systems, perhaps due to its stronger coordination with the metal atoms because of its strong oxophilic nature, smaller-sized donors, such as DEE, showed obvious poisoning effects on the active species and resulted in significantly decreased catalytic activities, whereas for other compounds, the polymer yields were hardly influenced. For DBE- and PME-involved systems, the molecular weights and dispersities in the obtained polymers were very close to the donor-free systems; nevertheless, for HO- and UO-involved counterparts, slightly higher molecular weights as well as broader dispersities were concluded, indicating again decreased chain transfer efficiencies. For nitrogen-containing compounds, it seemed that detrimental effects were always observed, no matter whether it was from the decreased catalytic activities for TEA-involved systems, or the increased molecular weights or dispersities observed in TOA, DIEA, DIBA and PEA-involved systems.

**Figure 3:**
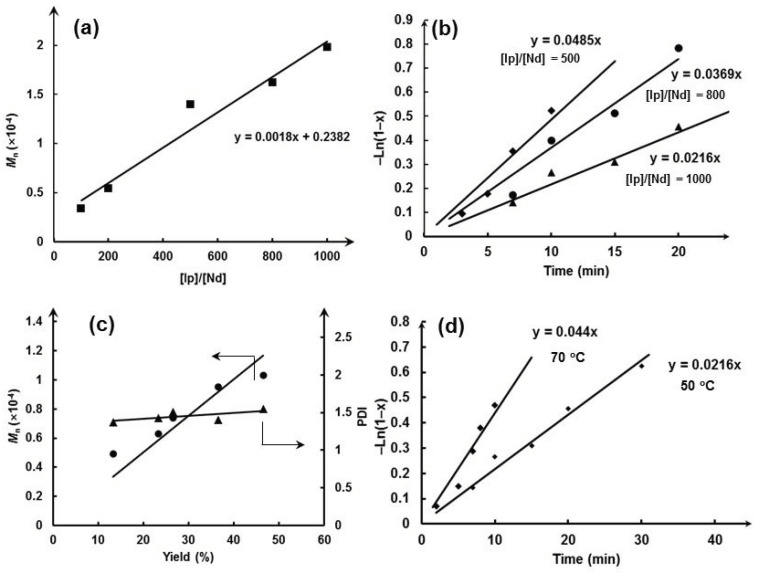
Plots on *M*_n_ against (Ip)/(Nd) (**a**); semilogarithmic plots on −ln(1 − *x*) against time at (Ip)/(Nd) = 500, 800, 1000 (**b**); plots on *M*_n_ and *M*_w_/*M*_n_ against polymer yields at (Ip)/(Nd) = 1000 (**c**); semilogarithmic plots on −ln(1 − *x*) against time at 50 °C and 70 °C (**d**).

A detailed kinetic study was also carried out in order to better elucidate the influence of external donors on the whole polymerization process. Through varying the (Ip)/(Nd) ratios from 100 to 1000 (Table 5), the molecular weights of the resultant PIps gradually increased from 0.34 × 10^4^ to 1.94 × 10^4^ Dalton and, most importantly, a good linearity between the monomer concentration and molecular weights was observed (Figure 3a) and, simultaneously, the dispersities kept relatively narrow and stayed around 1.50. Such a linear relationship agreed well with the donor-free systems in our previous reports [33,34,47], hinting that well-controlled polymerization behaviors occur even in the presence of T*t*BP. Polymerization rate investigations at different (Ip)/(Nd) ratios of 1000, 800, and 500 revealed that with an increasing catalyst concentration, monotonously increased polymerization rates were observed as expected. As can be seen in the semilogarithmic plots on −ln(1 − *x*) (wherein *x* is the polymer yield) vs. time shown in Figure 3b, gradually increased apparent first-order rate constants *k*_app_s of 0.0216, 0.0369, and 0.0485 min^−1^ were obtained. During the kinetic investigations at (Ip)/(Nd) = 1000, a good linear relationship between the *M*_n_ and the polymer yields was also observed; meanwhile, a narrow molecular weight distribution was kept (Figure 3c), indicating that the active species were always kept at same concentrations, i.e., the CCTP characteristic could be maintained throughout the whole polymerization process. For the T*t*BP-involved CCTP system, the polymerization rates were also very sensitive to temperature. As can be seen in the semilogarithmic plots on −ln(1 − *x*) vs. time shown in Figure 3d, when the polymerization temperature rose from 50 to 70 °C, the apparent first-order rate constants *k*_app_s increased from 0.0216 to 0.044, indicating the enhanced catalytic activities.

**Figure 4:**
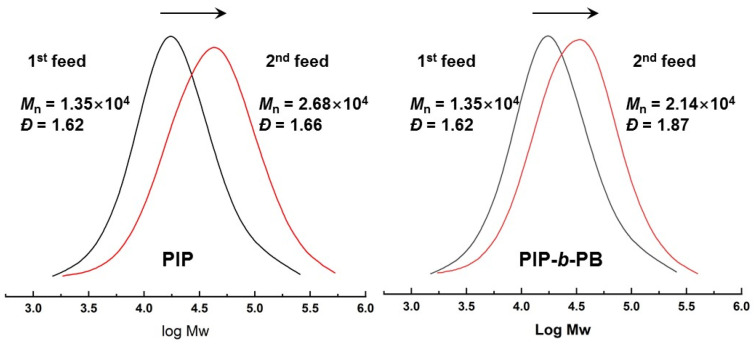
GPC profiles of the polymers obtained from seeding two-step polymerization by T*t*BP-involved CCTP.

**Figure 5:**
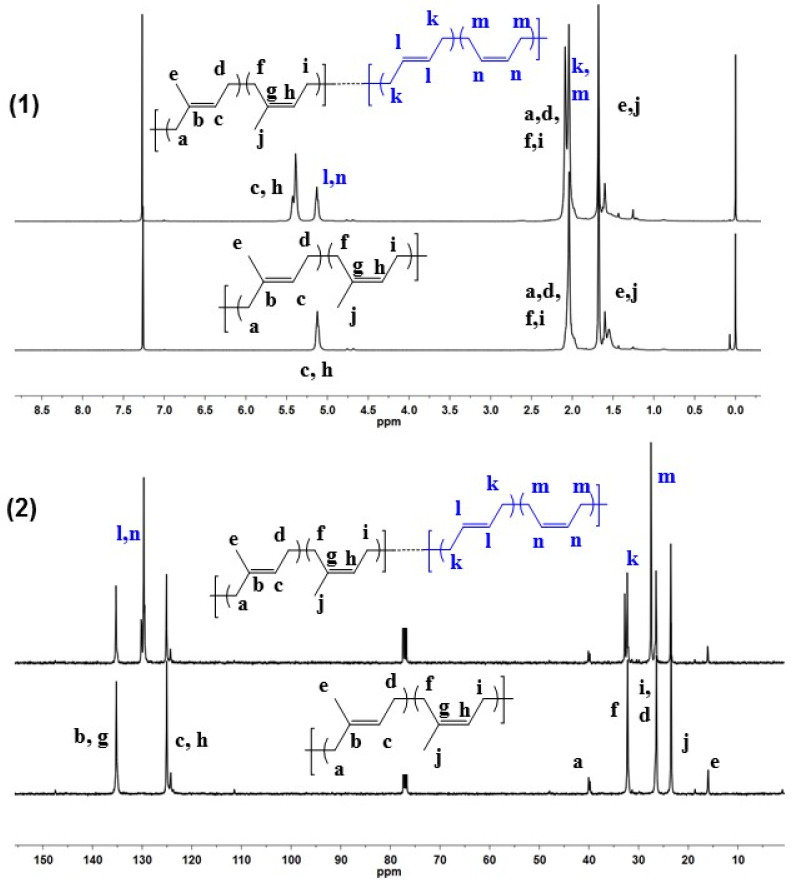
^1^H NMR (**1**) and ^13^C NMR (**2**) spectra of block copolymer of PIp-*b*-PB (minor 3,4 or 1,2 units were not assigned).

Inspired by the living nature of T*t*BP-involved CCTP system, seeding two-step polymerization by such a catalytic system was also examined. After the first polymerization step ((Ip)/(Nd) = 400) was completed within 4 h to give PIp with a molecular weight of 1.35 × 10^4^, the second feed of BD or Ip monomers with a ratio of (Ip)/(Nd) ((BD)/(Nd)) = 400 was added to the same system. It was found that the polymerizations could be continued smoothly, eventually resulting in PIp with nearly doubled molecular weights (2.68 × 10^4^) or the PIp-*b*-PB diblock copolymer with a molecular weight of 2.14 × 10^4^ (Figure 4). Narrow molecular weight distributions could be maintained after the second feeding, suggesting again the well-controlled polymerization behaviors for the T*t*BP-involved CCTP system. The obtained block copolymer PIp-*b*-PB was further well characterized by ^1^H and ^13^C NMR, from which the resonance peaks for the PIp and PB sequences could be clearly observed (Figure 5). For the ^13^C NMR, signals at 15.9 (e), 23.4 (j), 26.4 (i,d), 32.2 (f), 40.0 (a), 124.2 (c), 125.0 (h) and 135.2 (b,g) ppm were assigned to the polyisoprene units, and signals at 27.4 (m), 32.8 (k), 129.6 (l), and 130.1 (n) ppm were assigned to the polybutadiene units.

## 3. Materials and Methods

**Materials.** Nd(vers)_3_ (NdV) was a commercial product and diluted to 0.3 mol/L using hexane. Diisobutylaluminum hydride (DIBAH, Al(i-Bu)_2_H) was purchased from Akzo Nobel and diluted with hexane into a 2.0 mol/L solution. Me_2_SiCl_2_ was purchased from Energy Chemical Company and diluted with *n*-hexane to 0.20 mol/L. Isoprene (Ip) was purchased from Energy Chemical Company (shanghai, China) and dried by CaH_2_ via distillation under reduced pressure. In addition, 1,3-Butadiene was supplied by Ludong Chemical Corporation and purified by passing it through four columns packed with 4 Å and KOH prior to use. All the external donors used herein, including tri(*n*-butyl)phosphine (T*n*BP, 95%), tri(*tert*-butyl)phosphine (T*t*BP, 96%), tri(*n*-octyl)phosphine (TOP, 97%), triphenylphosphine (TPP, 98%), diphenylmethylphosphine (DPMP, 98%), diethylether (DEE, HPLC grade), dibutylether (DBE, 99%), phenylmethylether (PME, AR grade), 1-hexanol (HO, 99%), undec-10-en-1-ol (UO, 96%), triethylamine (TEA, HPLC grade), trioctylamine (TOA, 96%), diisopropylethylamine (DIEA, 97%), diisobutylamine (DIBA, 96%), and phenylethylamine (PEA, 97%), were purchased from Energy Chemical Company, and diluted with *n*-hexane before use (for TPP, HO, and UO, toluene was used as a solvent). Hexane or toluene was dried by heating to reflux over sodium/diphenylketyl until the solution turned blue and then distilled prior to use.

**Analytic methods.** The number-average molecular weight (*M*_n_) and molecular weight distribution (*M*_w_/*M*_n_) of the polymers were measured at 40 °C, using gel permeation chromatography (GPC) equipped with a Waters 515 HPLC pump, four columns (HMW 7 THF, HMW 6E THF × 2, HMW 2 THF), and a Waters 2414 refractive index detector. Tetrahydrofuran (THF) was used as an eluent at a flow rate of 1.0 mL/min. Sample solutions were filtered through a 0.45 μm microfilter before injection. The values of *M*_n_ and *M*_w_/*M*_n_ were calculated using polystyrene (PS) calibration. A Nicolet iS10 infrared spectrometer produced by Thermo Company was used for testing. Film samples were prepared on a KBr pallet by casting a carbon disulfide solution (*ca*. 2–8 mg/mL) of the polymer. The 1,4- and 3,4- contents were calculated according to a previous report [48].

The ratios of the 1,4 and 3,4 structures in the isoprene unit, from the absorption bands at 836 and 890 cm^−1^, employed the following formulas:

1,4 content(%) = 100 × (145 × A_836_ − 1.95 × A_890_)/C

3,4 content(%) = 100 × (19.9 × A_890_ − 1.79 × A_836_)/C

wherein C = (145 × A_836_ − 1.95 × A_890_) + (19.9 × A_890_ − 1.79 × A_836_), and A_836_ and A_890_ are the absorbances at 836 and 890 cm^−1^, respectively.

The microstructures of the obtained polyisoprene were further confirmed by the ^1^H NMR spectrum, according to the following method:

1,4 content(%) = *I*_5.10–5.20_/(*I*_5.10–5.20_ + *I*_4.69–4.77_)

3,4 content(%) = *I*_4.69–4.77_/(*I*_5.10–5.20_ + *I*_4.69–4.77_)

wherein, *I*_4.69–4.77_ and *I*_5.10–5.20_ is the integral area of the peaks at 4.69–4.77 ppm and 5.10–5.20 ppm, respectively.

**Catalysts preparation during the aging stage.** For the typical donor-involved CCTP system, the catalyst was prepared during the aging state according to procedure 1: into a 20 mL ampoule typical amounts of Nd(vers)_3_, BD, Al(iBu)_2_H, and external donors in the predesigned ratios were sequentially added, and the rection mixture was left to be aged at 50 °C for 20 min. Afterwards, a hexane solution of SiMe_2_Cl_2_ was added, and the reaction mixture was further aged for 30 min to obtain a yellow-green homogeneous catalyst, which was added to the external donors and, then, stored prior to use. For the catalyst addition order studies, premixing of the donors with Nd(vers)_3_ (procedure 2) or Al(iBu)_2_H (procedure 3) was performed in advance, before reacting with the other components. The catalyst addition order is also illustrated in Figure 2 to avoid confusion.

**Polymerization procedure.** A hexane solution of isoprene was placed in an oxygen- and moisture-free 50 mL ampule bottle with a rubber septum. After the solution was brought to the desired temperature, the prepared catalyst solution and external donors were sequentially added. Polymerization was carried out at 50 °C for 4 h and quenched by adding 2.0 mL of acidified ethanol, containing 2,6-di-*tert*-butyl-4-methylphenol as a stabilizer. The polymer was washed with ethanol repeatedly, and then dried in a vacuum at 40 °C to a constant weight. For the two-step seeding polymerizations to prepare the diblock copolymers, firstly, the living polyisoprene reaction system was prepared as described above. And then, the second feed of isoprene or 1,3-butadiene monomer solutions was added into the same polymerization, which was then left to react for another 5 h. The precipitation and drying procedures for the block copolymer are the same as that of polyisoprene preparation.

## 4. Conclusions

In this report, we carried out a systematic evaluation of the influence of external donors on the overall polymerization behaviors of the Nd-mediated CCTP of isoprene. Such research was mainly inspired by the need to maintain the CCTP characteristic even in the presence of donors, through which high atom economic property could be retained to achieve the goal of reducing the precatalyst cost. It was found that the incorporation of donors, such as T*t*BP, caused a negative influence on the chain transfer efficiencies, and resulted in gradually decreased atom economies, as revealed from the monotonously decreased *N*_p_ values. Nevertheless, when the ratio of the T*t*BP and Nd precatalyst was lower than 0.5, such donor-involved system could reveal a typical CCTP characteristic, implying a critical ratio of 0.5 if a high atom economy was targeted. Such an outcome is very instructive for the continuous industrial production of NdBR and NdIR because the donor concentration much be reduced to a certain concentration. Mechanistic studies showed that the decreased transfer efficiency mainly originated from the coordination of the donors with the Nd precatalyst, rather than the alkylaluminium CTA, implying that changing the microenvironment around the Nd center is more important in governing the chain transfer reactions. Additionally, the present research also concluded that introduction of the 0.01 donor had a negligible influence on the CCTP performance, and high atom economic values as in donor-free systems were obtained. Under such a donor concentration, other polymerization conditions, including (Al)/(Nd) ratios and (Cl)/(Nd) ratios, were also optimized. More importantly, under such an optimal condition, the polymerization proceeded in a well-controlled manner, as revealed by the good linear relationships between the *M*_n_ of the PIps against the polymer yields, as well as between the *M*_n_ against the (IP)/(Nd) ratios. A detailed kinetic investigation also showed that the polymerization rates were proportional to the catalyst concentrations, as well as the polymerization temperatures. With the aid of such well-controlled behavior, the present donor-involved CCTP system also allowed for seeding two-step polymerizations to prepare diblock copolymers with precisely controlled molecular weights. Expanding the types of donors to more phosphine, oxygen, and nitrogen-containing compounds showed that they also affected the CCTP behaviors depending on their steric and electronic properties. In summary, the present research presents a whole map of the donor-involved CCTP system. In contrast, most of other reports that are mainly focused on searching for suitable precatalysts or CTAs, the present study shifts the attention to external factors for the first time. Although decreased chain transfer efficiencies are witnessed, it still provides instructive information for designing future CCTP systems and, in particular, provides significant information on practical application for the continuous industrial production of NdBR and NdIR.

## Figures and Tables

**Table 1:** CCTP of Ip under different concentrations of T*t*BP ^a^.

Entry	(D)/(Nd)	Yield (wt %)	*M*_n_ × 10^−4 b^	*M*_w_/*M*_n_ ^b^	Microstructure (%) ^c^		*N*_p_ ^d^
					1,4	3,4	
1	0	100	1.85	1.61	97.0	3.0	3.67
2	0.01	100	1.94	1.66	96.8	3.2	3.50
3	0.1	100	2.01	1.61	96.1	3.9	3.38
4	0.5	100	2.53	1.70	96.5	3.5	2.69
5	1	100	3.12	2.45	97.3	2.7	2.18
6	3	100	3.69	2.95	98.0	2.0	1.84

^a^ Polymerization conditions: (Ip) = 1.5 M, (Ip)/(Nd) = 1000, (Cl)/(Nd) = 2.0, (Al)/(Nd) = 25, hexane, 50 °C, 4 h. ^b^ Determined by GPC using polystyrene standards. ^c^ Determined by IR and NMR spectra (see representative ^1^H NMR and FTIR spectra in Figures S1–S4 in supplementary file). ^d^ The number of polymer chains produced per Nd atom, *N*_p_ = (Ip)/(Nd) × 68.12 × Yield/*M*_n_.

**Table 2:** Influence of the addition order of the catalyst components on CCTP behaviors ^a^.

Entry	Aging Order	Yield (wt %)	*M*_n_ × 10^−4 b^	*M*_w_/*M*_n_ ^b^	Microstructure (%) ^c^		*N*_p_ ^d^
					1,4	3,4	
1	(Nd + BD + Al) → P → Cl	100	1.94	1.66	96.8	3.2	3.50
2	(Nd + P) → BD → Al → Cl	100	1.95	1.73	96.9	3.1	3.49
3	Nd → BD → (Al + P) → Cl	100	2.21	1.76	96.1	3.9	3.08

^a^ Polymerization conditions: (IP) = 1.5 M, (IP)/(Nd) = 1000, (Cl)/(Nd) = 2.0, (Al)/(Nd) = 25, hexane, 50 °C, 4 h. ^b^ Determined by GPC using polystyrene standards. ^c^ Determined by IR and NMR spectra. ^d^ The number of polymer chains produced per Nd atom, *N*_p_ = (Ip)/(Nd) × 68.12 × Yield/*M*_n._

**Table 3:** CCTP of Ip under different conditions in the presence of T*t*BP.

Entry ^a^	(Al)/(Nd)	(Cl)/(Nd)	Yield (wt %)	*M*_n_^b^ × 10^−4 b^	*M*_w_/*M*_n_ ^b^	Microstructure (%) ^c^	
						1,4	3,4
1	10	2.0	-	-	-	-	-
2	15	2.0	71	5.24	2.46	96.5	3.5
3	20	2.0	80	4.22	2.90	96.8	3.2
4	25	2.0	100	1.94	1.66	96.8	3.2
5	30	2.0	100	1.53	1.74	96.9	3.1
6	25	0.5	60	0.45	2.06	96.0	4.0
7	25	1.0	73	1.16	1.64	96.7	3.3
8	25	3.0	100	2.95	1.80	97.0	3.0
9	25	5.0	97	2.31	1.70	97.1	2.9

^a^ Polymerization conditions: (Ip) = 1.5 M, (Ip)/(Nd) = 1000, (D)/(Nd) = 0.01, hexane, 50 °C, 4 h. ^b^ Determined by GPC using polystyrene standards. ^c^ Determined by IR and NMR spectra.

**Table 4:** CCTP of Ip in the presence of different types of external donors.

Entry ^a^	D	(D)/(Nd)	Yield (wt %)	*M*_n_^b^ × 10^−4 b^	*M*_w_/*M*_n_ ^b^	Microstructure (%) ^c^	
						1,4	3,4
1	-	-	100	1.85	1.61	97.0	3.0
2	P	T*n*BP	100	1.70	1.64	96.9	3.1
3		T*t*BP	100	1.94	1.66	96.8	3.2
4		TOP	100	1.71	1.65	97.1	2.9
5		TPP	100	1.88	1.75	93.9	6.1
6		DPMP	100	1.76	1.71	96.2	3.8
7	O	DEE	71	7.40	3.22	96.2	-
8		DBE	100	1.90	1.58	96.5	3.5
9		PME	100	1.86	1.66	96.0	4.0
10		HO	100	2.04	1.79	96.7	3.3
11		UO	100	2.13	1.63	96.4	3.6
12	N	TEA	70	1.94	1.67	96.6	3.4
13		TOA	100	2.03	1.75	96.8	3.2
14		DIEA	100	2.20	1.77	96.1	3.9
15		DIBA	100	2.09	1.95	96.9	3.1
16		PEA	100	1.86	1.78	96.0	4.0

^a^ Polymerization conditions: (Ip) = 1.5 M, (Ip)/(Nd) = 1000, (Donor)/(Nd) = 0.01, (Cl)/(Nd) = 2.0, (Al)/(Nd) = 25, hexane, 50 °C, 4 h. ^b^ Determined by GPC using polystyrene standards. ^c^ Determined by IR and NMR spectra.

**Table 5:** CCTP of IP with various (Ip)/(Nd) ratios in the presence of T*t*BP.

Entry ^a^	(Ip)/(Nd)	Yield (wt %)	*M*_n_^b^ × 10^−4 b^	*M*_w_/*M*_n_ ^b^	Microstructure (%) ^c^	
					1,4	3,4
1	100	100	0.34	1.45	95.6	3.4
2	200	100	0.54	1.54	96.1	3.9
3	500	100	1.40	1.63	96.9	3.1
4	800	100	1.62	1.50	97.0	3.0
5	1000	100	1.94	1.66	96.8	3.2

^a^ Polymerization conditions: (IP) = 1.5 M, (Donor)/(Nd) = 0.01, (Cl)/(Nd) = 2.0, (Al)/(Nd) = 25, hexane, 50 °C, 4 h. ^b^ Determined by GPC using polystyrene standards. ^c^ Determined by IR and NMR spectra.

## Data Availability

Data is unavailable due to privacy.

## Supplementary Materials

The following supporting information can be downloaded at: https://www.mdpi.com/article/10.3390/molecules28217364/s1. ^1^H NMR, FT-IR spectroscopies and DSC curves for representative polyisoprene products can be found in the supplementary files. Figure S1: ^1^H NMR spectrum of polyisoprene obtained from Table 1, entry 1; Figure S2: ^1^H NMR spectrum of polyisoprene obtained from Table 1, entry 2; Figure S3: FT-IR spectrum of polyisoprene obtained from Table 1, entry 1; Figure S4: FT-IR spectrum of polyisoprene obtained from Table 1, entry 2; Figure S5: DSC curve of polyisoprene obtained from Table 1, entry 2.
