# Supplementary material for: Neodymium-Mediated Coordinative Chain Transfer Polymerization of Isoprene in the Presence of External Donors
Source: Molecules. 2023 Oct 31;28(21):7364. doi: 10.3390/molecules28217364 (PMC10648742; doi:10.3390/molecules28217364)
Supplement: Supplementary file 1 [file molecules-28-07364-s001.zip › molecules-2642261-supplementary.pdf]

Supporting information

Neodymium-Mediated Coordinative Chain Transfer Polymerization of Isoprene in the Presence of External Donors

Aiwu Ding [1,2], Liang Fang [3,*], Chunyu Zhang [3], Heng Liu [3], Xuequan Zhang [3] and Jianhe Liao [4,*]

1 School of Life Sciences, Hainan University, Haikou 570228, China; dingaw@163.com

2 China Hainan Rubber Industry Group Co., Ltd., Haikou 570106, China

3 Key Laboratory of Rubber-Plastics, Ministry of Education, Shandong Provincial Key Laboratory of Rubber-Plastics, Qingdao University of Science & Technology, Qingdao 266061, China; cyzhang@qust.edu.cn (C.Z.); hengliu@qust.edu.cn (H.L.); xqzhang@qust.edu.cn (X.Z.)

4 School of Materials Science and Engineering, Hainan University, Haikou 570228, China

* Correspondence: bh190@qust.edu.cn (L.F.); 990359@hainanu.edu.cn (J.L.)

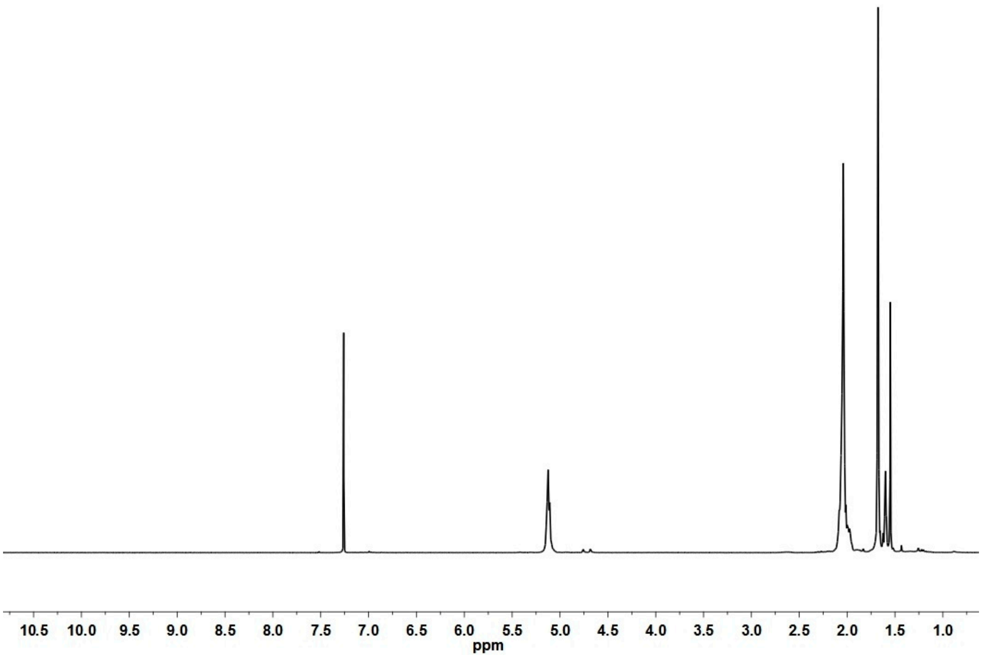

Figure S1. ^{1}H NMR spectrum of polyisoprene obtained from Table 1, entry 1.

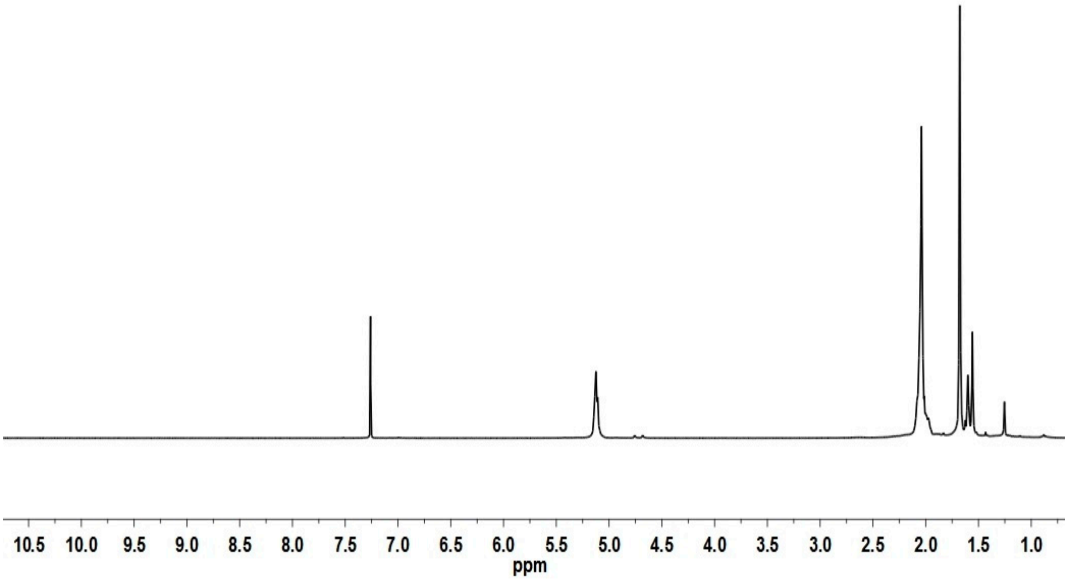

Figure S2. ^{1}H NMR spectrum of polyisoprene obtained from Table 1, entry 2.

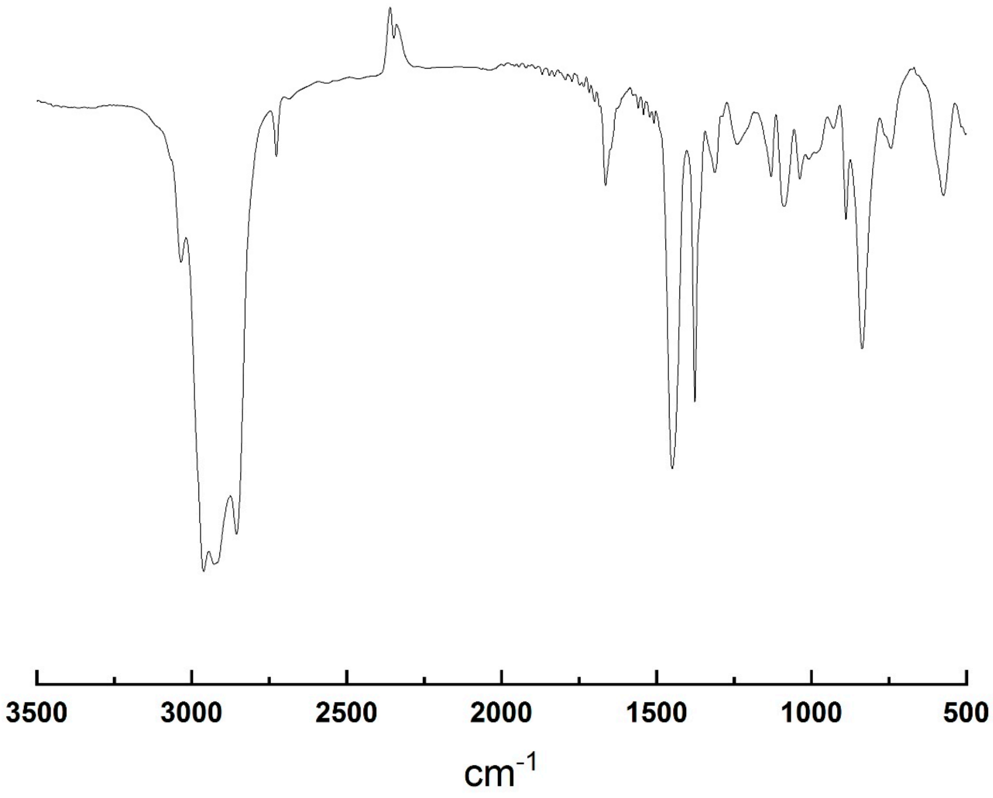

Figure S3. FT-IR spectrum of polyisoprene obtained from Table 1, entry 1.

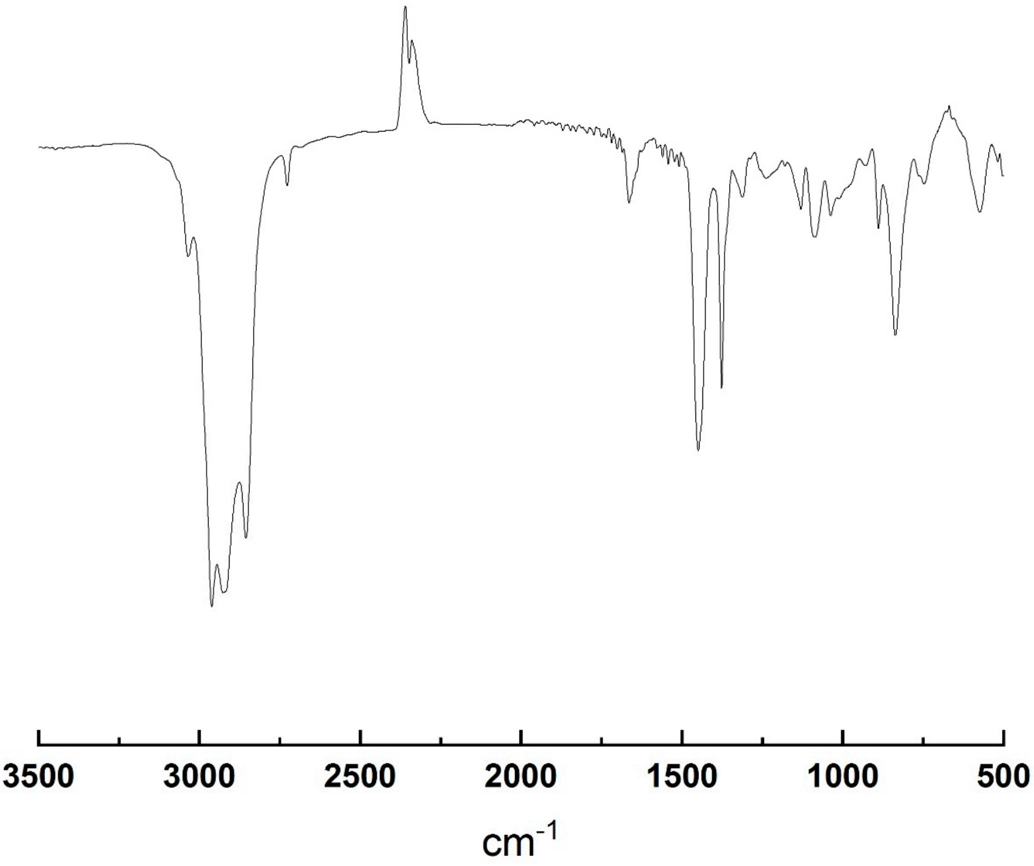

Figure S4. FT-IR spectrum of polyisoprene obtained from Table 1, entry 2.

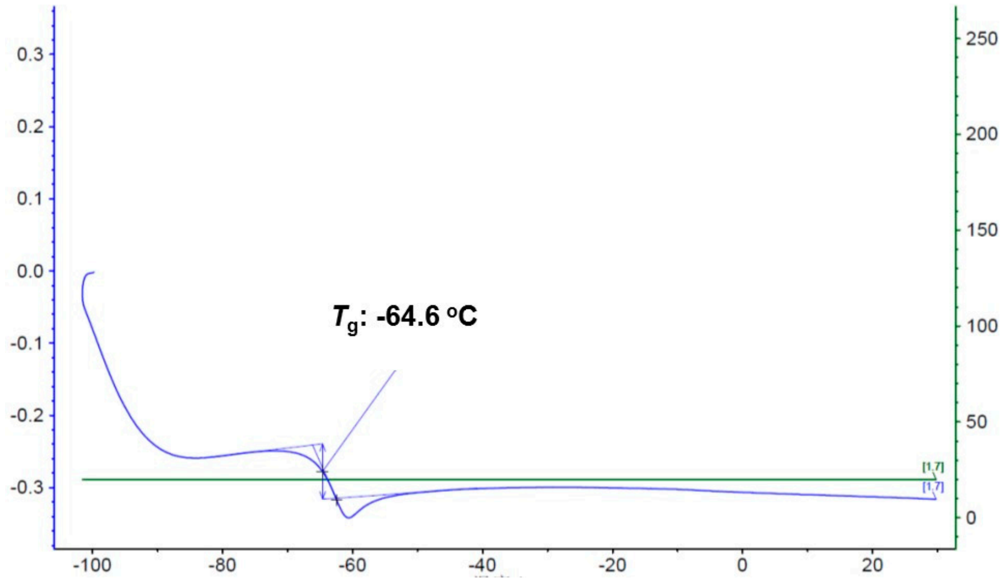

Figure S5. DSC curve of polyisoprene obtained from Table 1, entry 2.
